# In Silico Pharmacokinetics, ADMET Study and Conceptual DFT Analysis of Two Plant Cyclopeptides Isolated From Rosaceae as a Computational Peptidology Approach

**DOI:** 10.3389/fchem.2021.708364

**Published:** 2021-08-12

**Authors:** Norma Flores-Holguín, Juan Frau, Daniel Glossman-Mitnik

**Affiliations:** ^1^ Laboratorio Virtual NANOCOSMOS, Departamento de Medio Ambiente y Energía, Centro de Investigación en Materiales Avanzados, Chihuahua, Mexico; ^2^ Departament de Química, Universitat de les Illes Balears, Palma de Mallorca, Spain

**Keywords:** plant cyclopeptides, pharmacokinetics, ADMET, conceptual density functional theory, Koopmans in density functional theory

## Abstract

This research presents the outcomes of a computational determination of the chemical reactivity and bioactivity properties of two plant cyclopeptides isolated from Rosaceae through the consideration of Computational Peptidology (CP), a protocol employed previously in the research of similar molecular systems. CP allows the prediction of the global and local descriptors that are the integral foundations of Conceptual Density Functional Theory (CDFT) and which could help in getting in the understanding of the chemical reactivity properties of the two plant cyclopeptides under study, hoping that they could be related to their bioactivity. The methodology based on the Koopmans in DFT (KID) approach and the MN12SX/Def2TZVP/H2O model chemistry has been successfully validated. Various Chemoinformatics tools have been used to improve the process of virtual screening, thus identifying some additional properties of these two plant cyclopeptides connected to their ability to behave as potentially useful drugs. With the further objective of analyzing their bioactivity, the CP protocol is complemented with the estimation of some useful parameters related to pharmacokinetics, their predicted biological targets, and the Absorption, Distribution, Metabolism, Excretion and Toxicity (ADMET) parameters related to the bioavailability of the two plant cyclopeptides under study are also reported.

## 1 Introduction

Plant-based bioactive compounds have drawn attention of all communities around the world due to their unique biochemical activities and health benefits. Research studies have confirmed the safeguarding effects of certain plant-based diets on cardiovascular diseases, obesity, cancer, diabetes, etc. (Guha et al., 2021). Plant-based drugs from secondary metabolites constitute more than 25% of approved new drugs during last 30 years. Also, 50% of the commercially successful medicinal components were developed based on knowledge from plant secondary metabolites and their structures (Chaudhari and Chakraborti, 2021).

Bioactive peptides are organic substances formed by amino acids joined by covalent bonds known as amide or peptide bonds. Although peptides can exist free in terrestrial plants and marine sources, the vast majority of known bioactive peptides are enclosed within the structure of the proteins and can be released using enzymatic processes. Bioactive peptides play a significant role in human health by affecting the digestive, endocrine, cardiovascular, immune, and nervous systems. The increasing interest in bioactive peptides has incentivized the scientific community in the exploration and development of new therapeutic drugs based on these peptides (Sánchez and Vázquez, 2017).

Cyclic peptides can be considered as an alternative scaffold. The smaller size and several functional groups of peptides help to make the contact area large enough to provide good selectivity. Their ability to form several hydrogen bonds make easier to obtain strong binding affinity. Moreover, the cyclization of peptides helps in the generation of structural and functional features that are considered to be critical for their use as pharmaceutical drugs, including resistance to degradation by blood proteases. Also, the cyclization of the peptides facilitates the passage through the cell membrane. Because of such favorable features, many cyclic peptides from terrestrial plant and marine sources and their derivatives have been considered for drug design and development (Gang et al., 2018). Besides these biological features, cyclopeptides have smaller sizes than proteins and reduced flexibilities, exhibiting lower conformations than their linear counterparts, thus making easier and affordable the DFT calculations of their structures and properties.

By considering that the knowledge of the chemical reactivity properties of a given molecule is essential for the development of new therapeutic drugs, we are currently researching on new families of cyclopeptides obtained from terrestrial plants and marine sources hoping that the obtained information could be of help for the design of pharmaceutical based on these peptides (Kim, 2013). The objective of the present work is to report the global and local chemical reactivity descriptors of two plant cyclopeptides, Pashinintides A and B, that have been isolated from Rosacea (Cai et al., 2014) by making use of the Conceptual DFT (CDFT) methodology (Parr and Yang, 1989; Chermette, 1999; Geerlings et al., 2003; Toro-Labbé, 2007; Chattaraj, 2009; Geerlings et al., 2020; Chakraborty and Chattaraj, 2021). A recent review has highlighted the basic electronic structure principles and various reactivity descriptors defined within the premise of CDFT (Chakraborty and Chattaraj, 2021). The study is complemented by considering the report of some additional properties of potential application in Structure Activity Relationships (SAR) research for the development of therapeutic drugs, and also with the bioactivity radars related to the drug-like behavior of the studied peptides, their predicted biochemical targets and the values associated with Pharmacokinetics and ADMET properties (Daina et al., 2017; Pires et al., 2015; Daina et al., 2019) through standard Chemoinformatics procedures (Begam and Kumar, 2012; González-Medina et al., 2017). By considering this integrative strategy, called Conceptual DFT-based Computational Peptidology as a branch of Computational Chemistry dedicated to the study of peptides and cyclopeptides, the current research represents an extension of our recent studies on the properties of some families of therapeutic peptides of marine origin (Frau et al., 2018; Flores-Holguín et al., 2019a; Frau et al., 2019; Flores-Holguín et al., 2019c; Flores-Holguín et al., 2020; Flores-Holguín et al. 2020a; Flores-Holguín et al., 2020b; Flores-Holguín et al., 2021).

## 2 Materials and Methods

### 2.1 In Silico Pharmacokinetics Analysis and Absorption, Distribution, Metabolism, Excretion and Toxicity Study

**FIGURE 1:**
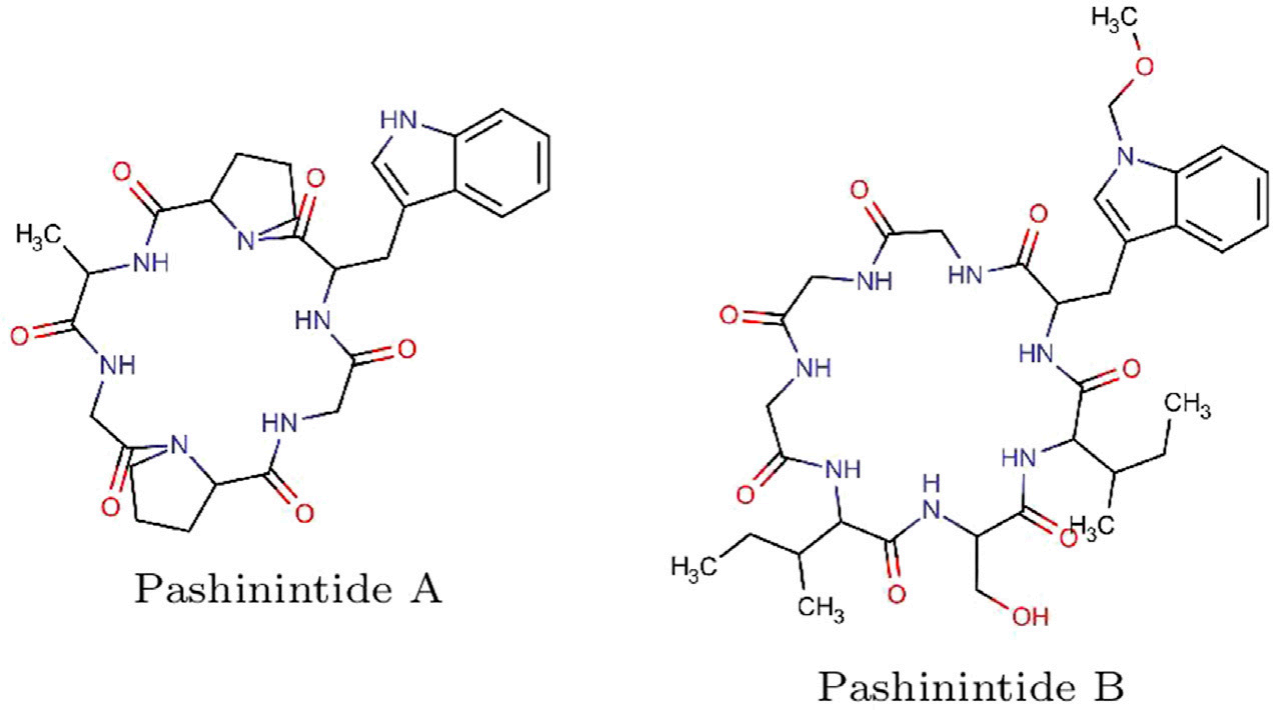
Graphical sketches of the molecular structures of the Pashinintides A and B plant cyclopeptides.

The starting molecular structures of the two plant cyclopeptides to be studied, shown in Figure 1, lwere obtained from PubChem (https://pubchem.ncbi.nlm.nih.gov), which is an open chemistry database.

As a first step, the SMILES (Simplified Molecular Input Line Entry Specification) notation of every studied compound, which was obtained by accessing ChemDoodle 11.3.0 software, was fed into the online program Chemicalize, a software developed by ChemAxon (http://www.chemaxon.com), which was used for naming, molecular finger prints, structure generation and the prediction of several properties related to Chemoinformatics (http://chemicalize.com/) (accessed March 2021).

The similarity searches in the chemical space of compounds with molecular structures similar to those that are being studied was accomplished using the online available Molinspiration software from Molinspiration Cheminoformatics (https://www.molinspiration.com/) (accessed, March 2021) which was used for the prediction of the bioactivity scores for different drug targets.

A Webtool named SwissTargetPrediction for efficient prediction of protein targets of small molecules has been considered for the determination of the potential bioactivity of the two terrestrial plant cyclopeptides considered in this study (Daina et al., 2019). The associated website allows the estimation of the most probable macromolecular targets of a small molecule, assumed as bioactive. During the process of development of a new therapeutic drug, it is of the most importance to adcquire a knowledge of the fate of the pharmacokinetics, that is, the fate of a compound in the organism. This is usually performed by through individual indices that are called Absorption, Distribution, Metabolism, Excretion and Toxicity (ADMET) parameters. These parameters are generally obtained using computer models as an alternative to the experimental procedures for their determination. In this research, some ADME parameters were estimated with the aid of Chemicalize and the online available SwissADME software (Daina et al., 2017).Additional information about the Pharmacokinetics parameters and the ADMET properties were obtained by resorting to pkCSM (Pires et al., 2015), a software for the prediction of small-molecule pharmacokinetic properties using SMILES (https://biosig.unimelb.edu.au/pkcsm/) (accessed, March 2021).

### 2.2 Density Functional Theory Calculations

The goodness of a given density functional can be determined through a comparison of the results that it renders with the experimental values or with the results that can be obtained by means of high-level calculations. However, the lack of experimental results for the molecular systems under study or the large size of the molecules that made computationally impractical the use of some accurate methodologies. Kohn-Sham (KS) methodology includes the determination of the molecular energy, the electronic density and the orbital energies of a given system, related to the frontier orbitals including the Highest Occupied Molecular Orbital (HOMO) and Lowest Unoccupied Molecular Orbital (LUMO) (Young, 2001; Lewars, 2003; Cramer 2004; Jensen, 2007). This methodology is convenient when thinking of quantitative qualities related with Conceptual DFT descriptors (Parr and Yang, 1989; Chermette, 1999; Geerlings et al., 2003; Toro-Labbé, 2007; Chattaraj, 2009, Geerlings et al., 2020; Chakraborty and Chattaraj, 2021). Range-separated (RS) exchange-correlation density functionals are of extraordinary concern in Kohn-Sham DFT calculations (Iikura et al., 2001; Yanai et al., 2004; Heyd and Scuseria, 2004; Chai and Head-Gordon, 2008; Stein et al., 2009a; Stein et al., 2009b; Stein et al., 2010; Karolewski et al., 2011; Kuritz et al., 2011; Ansbacher et al., 2012; Kronik et al., 2012; Stein et al., 2012). A methodology called KID (Koopmans in DFT) has been established by our research group (Frau et al., 2018; Flores-Holguín et al., 2019a; Frau et al., 2019; Flores-Holguín et al., 2019c; Flores-Holguín et al., 2020; Flores-Holguín et al., 2020a; Flores-Holguín et al., 2020b; Flores-Holguín et al., 2021), t for the validation of a given density functional in terms of its internal coherence. Several descriptors associated with the results of the HOMO and LUMO calculations are related to the results obtained from the estimation of the vertical I and A following the ΔSCF procedure, where SCF refers to the Self-Consistent Field technique. It has been demonstrated that there is a relationship between the KID descriptors and the Koopmans’ theorem or the Ionization Energy theorem, which is its equivalent within the Generalized Kohn-Sham (GKS) version of DFT, by connecting *ϵ*_*H*_ to -I, *ϵ*_*L*_ to -A, and their actions by defining the HOMO – LUMO gap *J*_*I*_ = |*ϵ*_*H*_ + *E*_*gs*_(*N* − 1) − *E*_*gs*_(*N*)|, *J*_*A*_ = |*ϵ*_*L*_ + *E*_*gs*_(*N*) − *E*_*gs*_(*N* + 1)|, and $J_{HL}=\sqrt{{J_{I}}^{2}+{J_{A}}^{2}}$. It should be noticed that the *J*_*A*_ descriptor consists of an approximation which is only valid if the HOMO of the radical anion (the SOMO) resembles the LUMO of the neutral system. For this reason, another descriptor ΔSL has been designed by our research group (Frau et al., 2018; Flores-Holguín et al., 2019a; Frau et al., 2019; Flores-Holguín et al., 2019c; Flores-Holguín et al., 2020; Flores-Holguín et al. 2020a; Flores-Holguín et al., 2020b; Flores-Holguín et al., 2021), to help in the verification of the accuracy of the approximation.

Taking into account the KID methodology considered in the previous research being integrated into the finite difference approximation (Frau et al., 2018; Flores-Holguín et al., 2019a; Frau et al., 2019; Flores-Holguín et al., 2019c; Flores-Holguín et al., 2020; Flores-Holguín et al., 2020a; Flores-Holguín et al., 2020b; Flores-Holguín et al., 2021), the following definitions can be used for the global descriptors that help in the understanding of the chemical reactivity of the molecular systems (Parr and Yang, 1989; Chermette, 1999; Geerlings et al., 2003; Gázquez et al., 2007; Chattaraj et al., 2009; Chakraborty and Chattaraj, 2021): Electronegativity as $\chi =-\frac{1}{2}\left(I+A\right)\approx \frac{1}{2}\left(\epsilon_{L}+\epsilon_{H}\right)$, Global Hardness as *η* = (*I* − *A*) ≈ (*ϵ*_*L*_ −*ϵ*_*H*_), Electrophilicity as *ω* = $\mu^{2}/2\eta ={\left(I+A\right)}^{2}/4\left(I-A\right)\approx {\left(\epsilon_{L}+\epsilon_{H}\right)}^{2}/4\left(\epsilon_{L}-\epsilon_{H}\right)$, Electrodonating Power as *ω* ^−^ = ${\left(3I+A\right)}^{2}/16\left(I-A\right)\approx {\left(3\epsilon_{H}+\epsilon_{L}\right)}^{2}/16\eta$, Electroaccepting Power as *ω* ^+^ = ${\left(I+3\;A\right)}^{2}/16\left(I-A\right)\approx {\left(\epsilon_{H}+3\epsilon_{L}\right)}^{2}/16\eta$ and Net Electrophilicity as Δ*ω* ^±^ = *ω* ^+^ − (−*ω* ^−^) = *ω* ^+^ + *ω* ^−^, being *ϵ*_*H*_ and *ϵ*_*L*_ the HOMO and LUMO energies associated with each of the peptides considered in this work. It is worth to mention that for the global indices the chemical power is directly related with the electronic density as well as the corresponding Hohenberg-Kohn functional (Putz, 2011).

As a complement of these global reactivity descriptors that arise from Conceptual DFT (Parr and Yang, 1989; Chermette, 1999; Geerlings et al., 2003; Gázquez et al., 2007; Chattaraj et al., 2009; Chakraborty and Chattaraj, 2021), Domingo and his collaborators (Domingo et al., 2008; Jaramillo et al., 2008; Domingo and Sáez, 2009; Domingo and Perez, 2011; Domingo et al., 2016) have proposed a Nucleophilicity index N through the consideration of the HOMO energy obtained through the KS scheme with an arbitrary shift of the origin taking the molecule of tetracyanoethylene (TCE) as a reference.

The determination of the conformers of the molecules considered in the current study was performed by resorting to MarvinView 17.15 available from ChemAxon (http://www.chemaxon.com) by doing Molecular Mechanics calculations through the overall MMFF94 force field (Halgren, 1996a, Halgren, 1996b, Halgren, 1999; Halgren and Nachbar, 1996; Halgren, 1996c). This was followed by a geometry optimization and frequency calculation by means of the Density Functional Tight Binding (DFTBA) methodology (Frisch et al., 2016). This last step was required for the verification of the absence of imaginary frequencies as a check for the stability of the optimized structures as being a minimum in the energy landscape. The electronic properties and the chemical reactivity descriptors of the studied molecules involved the use of MN12SX/Def2TZVP/H2O model chemistry (Weigend and Ahlrichs, 2005; Weigend, 2006; Peverati and Truhlar, 2012) on the optimized molecular structures due to is ability in the verification of the “Koopmans in DFT” (KID) protocol (Frau and Glossman-Mitnik, 2018a; Frau and Glossman-Mitnik, 2018b; Frau and Glossman-Mitnik, 2018c; Frau and Glossman-Mitnik, 2018d; Frau and Glossman-Mitnik, 2018e; Frau and Glossman-Mitnik, 2018f; Flores-Holguín et al., 2019a; Flores-Holguín et al., 2019b; Flores-Holguín et al., 2019d; Frau et al., 2019; Flores-Holguín et al., 2019c, Flores-Holguín et al., 2020; Flores-Holguín et al., 2020a; Flores-Holguín et al., 2020b; Flores-Holguín et al., 2021) using Gaussian 16 (Frisch et al., 2016) and the SMD model for the simulation of the solvent (Marenich et al., 2009). This model chemistry considers the MN12SX screened-exchange density functional (Peverati and Truhlar, 2012) together with the Def2TZVP basis set (Weigend and Ahlrichs, 2005; Weigend, 2006) and in all cases the charge of the molecules is equal to zero while the radical anion and cation have been considered in the doublet spin state.

## 3 Results and Discussion

### 3.1 Physicochemical Properties, Bioactivity Scores and Biological Targets

The names, identifiers, molecular fingerprints and basic properties of the two Pashinintides A and B plant cyclopeptides are presented in Table 1, while their geometrical and structural properties are displayed in Table 2.

**TABLE 1:** Names, identifiers, molecular fingerprints and basic properties of the studied molecular systems.

Property	Value
Common name	Pashinintide A
PubChem CID	122386973
Molar mass	565.631 g/mol
Exact mass	565.264881875 Da
Formula	C_28_H_35_N_7_O_6_
Composition	C (59.46%), H (6.24%), N (17.33%), O (16.97%)
IUPAC name	3-[(1H-indol-3-yl)methyl]-18-methyl-1,4,7,13,16,19-hexaazatricyclo[19.3.0.0^9^,^13^] tetracosane-2,5,8,14,17,20-hexone
Traditional name	3-(1H-indol-3-ylmethyl)-18-methyl-1,4,7,13,16,19-hexaazatricyclo[19.3.0.0^9^,^13^] tetracosane-2,5,8,14,17,20-hexone
SMILES	CC1NC(=O)C2CCCN2C(=O)C(CC2=CNC3=CC=CC=C23)NC(=O)CNC(=O)
	C2CCCN2C(=O)CNC1=O
InChI	InChI=1/C28H35N7O6/c1-16-25(38)31-15-24(37)34-10-4-8-21(34)26(39)
	30-14-23(36)33-20(12-17-13-29-19-7-3-2-6-18(17)19)28(41)35-11-5-9-22
	(35)27(40)32-16/h2-3,6-7,13,16,20-22,29H,4-5,8-12,14-15H2,1H3,(H,30,39)
	(H,31,38)(H,32,40)(H,33,36)
InChIKey	MKXJIZUYLVDQCC-UHFFFAOYNA-N
IUPAC condensed	cyclo[Ala-Gly-Pro-Gly-Trp-Pro]
Common name	Pashinintide B
PubChem CID	122386974
Molar mass	714.821 g/mol
Exact mass	714.370075222 Da
Formula	C_34_H_50_N_8_O_9_
Composition	C (57.13%), H (7.05%), N (15.68%), O (20.14%)
IUPAC name	6,12-bis(butan-2-yl)-9-(hydroxymethyl)-3-[1-(methoxymethyl)-1H-indol-3-yl]
	methyl-1,4,7,10,13,16,19-heptaazacyclohenicosane-2,5,8,11,14,17,20-heptone
Traditional name	9-(hydroxymethyl)-3-[1-(methoxymethyl)indol-3-yl]methyl-6,12-bis(sec-butyl)
Traditional name	-1,4,7,10,13,16,19-heptaazacyclohenicosane-2,5,8,11,14,17,20-heptone
SMILES	CC1NC(=O)C2CCCN2C(=O)C(CC2=CNC3=CC=CC=C23)NC(=O)CNC(=O)
	C2CCCN2C(=O)CNC1=O
InChI	InChI=1/C28H35N7O6/c1-16-25(38)31-15-24(37)34-10-4-8-21(34)26(39)30-14
	23-23(36)33-20(12-17-13-29-19-7-3-2-6-18(17)19)28(41)35-11-5-9-22(35)27(40)
	32-16/h2-3,6-7,13,16,20-22,29H,4-5,8-12,14-15H2,1H3,(H,30,39)(H,31,38)
	(H,32,40)(H,33,36)
InChIKey	MKXJIZUYLVDQCC-UHFFFAOYNA-N
IUPAC condensed	cyclo[Gly-Gly-Gly-xiIle-Ser-xiIle-Trp(MeOMe)]

**TABLE 2:** Geometrical and structural properties of the studied molecular systems.

Property	Pashinintide A	Pashinintide B
Atom count	76	101
Non-hydrogen atom count	41	51
Asymmetric atom count	4	6
Rotatable atom count	2	9
Ring count	5	3
Aromatic ring count	2	2
Hetero ring count	4	2
FSP3	0.50	0.56
Hydrogen bond donor count	5	8
Hydrogen bond acceptor count	6	9
Formal charge	0	0
Van der Waals volume (Å^3^)	498.20	654.63
Van der Waals surface area (Å^2^)	797.07	1,071.58
Solvent accesible surface area (Å^2^)	688.91	729.09
Topological polar surface area (Å^2^)	172.81	238.09
Polarizability (Å^3^)	57.51	72.58
Molar refractivity (cm^3^/mol)	145.88	182.79

This information could it be of interest for future SAR studies based on these and other peptides as well as for potential derivatives designed for therapeutical purposes using Peptidomimetics.

**FIGURE 2:**
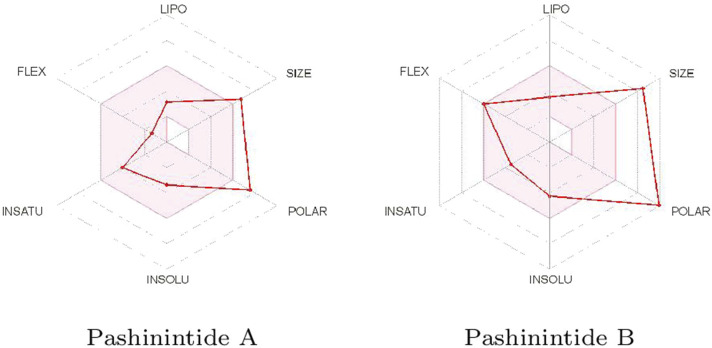
Bioavailability radars of the Pashinintides A and B.

The effect of the geometrical and structural properties on the bioavailability of the Pashinintides A and B presented in Table 2 can be better visualized considering the Bioavailability Radars displayed in Figure 2.

It can be appreciated that the two more important properties that could prevent the use of the Pashinintides A and B as therapeutic drugs are their molecular size and their polar character. Although these cyclic peptides violate some of the limits traditionally considered to be important for oral bioavailability of drug-like small molecules, it can be expected that the reduced flexibility could ease oral absorption (Nielsen et al., 2017). However, it must be remarked that these ideal values are based on the Lipinski’s Rule of Five (Lipinski et al., 2001), which is not always applicable to peptides (Zhang and Wilkinson, 2007; Doak et al., 2014; Santos et al., 2016; Nielsen et al., 2017; Sable et al., 2017).

The Bioactivity Scores for the Pashinintides A and B are shown in Table 3.

**TABLE 3:** Bioactivity scores for the Pashinintides A and B.

Property	Pashinintide A	Pashinintide B
GPCR Ligand	0.40	−0.54
Ion channel modulator	−0.20	−1.64
Nuclear receptor ligand	−0.03	−1.15
Kinase inhibitor	−0.20	−1.42
Protease inhibitor	0.53	−0.16
Enzyme inhibitor	0.04	−0.92

**FIGURE 3:**
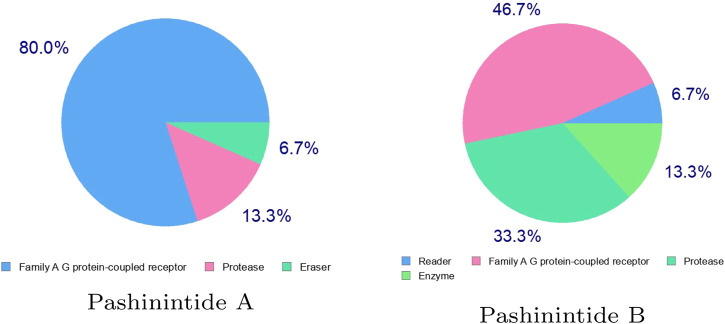
Predicted biological targets of the Pashinintides A and B.

It can be seen from the results on Table 3 that while the bioactivity of Pashinintide B towards the different targets is considered to be low, for Pashinintide A, its interactions as a GPCR Ligand and a Protease Inhibitors could be of importance for its consideration as a potential therapeutic drug. The same conclusion can be extracted by checking visually the predicted biological targets for these plant cyclopeptides shown in Figure 3.

### 3.2 Absorption, Distribution, Metabolism, Excretion and Toxicity Study

An ADMET study is the assessment of pharmacokinetics of a drug which stands for Absorption, Distribution, Metabolism, Excretion and Toxicity. The prediction of the fate of a drug and the effects caused by a drug inside the body, such as how much drug is absorbed if administered orally and how much is absorbed in the gastrointestinal tract, is an indispensable part of drug discovery. In a similar way, if the absorption is poor, its distribution and metabolism would be affected, which can lead to causing neurotoxicity and nephrotoxicity. Ultimately, the study is to understand the disposition of a drug molecule within an organism. Thus, ADMET study is one of the most essential parts of computational drug design.

#### 3.2.1 Absorption

A compound can reach a tissue, if it is taken into the bloodstream. Usually, a drug is administered often through mucous surfaces such as the digestive tract, i.e., intestinal absorption before it is taken up by the target cells. Factors like poor compound solubility, intestinal transit time, gastric emptying time, inability to permeate the intestinal wall and chemical instability in the stomach are responsible for reducing the extent of drug absorption after oral administration. Critically, absorption determines the bioavailability of a compound. Drugs with poor absorption are less desirable for oral administration, such as by inhalation or intravenously (Pires et al., 2015; Jujjavarapu et al., 2019).

The computed absorption properties of the Pashinintides A and B are presented in Table 4.

**TABLE 4:** Absorption properties of the Pashinintides A and B.

Property	Pashinintide A	Pashinintide B
Water solubility	−3.197	−3.377
Caco-2 permeability	0.814	0.803
Intestinal absorption	52.552	31.004
Skin permeability	−2.736	−2.735
P-glycoprotein substrate	Yes	Yes
P-glycoprotein I inhibitor	No	Yes
P-glycoprotein II inhibitor	No	No

The water solubility of a compound (logS) reflects the solubility of the molecule in water at 25°C. The predicted water solubility of a compound is given as the logarithm of the molar concentration (log mol/L) being their values very similar for both cyclopeptides. A compound is considered lo have a high Caco-2 permeability has a Papp >8 × 10^8^ cm/s. Thus, high Caco-2 permeability would translate in predicted values >0.90, presenting the Pashinintides A and B values which are a bit lower than the ideal one. The Intestine is normally the primary site for absorption of a drug from an orally administered solution. A molecule with an Intestinal Absorption of less than 30% is considered lo be poorly absorbed. From Table 4, both plant cyclopeptides will be highly absorbed. The P-glycoprotein has the function of a biological barrier by extruding toxins out of cells. The model predicts whether a given compound is likely to be a substrate of P-glycoprotein or not. The prediction is in the positive direction in both cases. Thus, the study predicts that both cyclopeptides will not act as P-glycoprotein II inhibitors, but Pashinintide A will not be a P-glycoprotein I inhibitor while Pashinintide B is likely to act in that way. Also, it can be predicted whether a given compound is likely to be skin permeable. A compound is considered to have a relatively low skin permeability if it has a log Kp > −2.5. It means that both cyclopeptides could be of interest for the development of transdermal drug delivery (Pires et al. (2015)).

#### 3.2.2 Distribution

The computed distribution properties of the Pashinintides A and B are presented in Table 5.

**TABLE 5:** Distribution properties of the Pashinintides A and B.

Property	Pashinintide A	Pashinintide B
VD	−0.055	−0.769
Fraction unbound	0.394	0.364
BBB permeability	−0.299	−0.789
CNS permeability	−3.921	−4.459

VD is the theoretical volume required by a drug to be uniformly distributed in blood. The higher the VD is, the more of a drug is distributed in tissue rather than plasma. From Table 5 and the usual standards, it can be said that VD for Pashinintide A is low and it is high for Pashinintide B. The Fraction Unbound parameter predicts the fraction that will be unbound in plasma resulting in the values shown in Table 5. The knowledge of the ability of a drug to cross into the brain is an important parameter that may help to reduce side effects and toxicities. A logBBB (for Blood-Brain Barrier) >−0.3 for a given drug is considered to easily cross the BBB while molecules with logBBB >−1 are poorly distributed to the brain, being predicted that both cyclopeptides have a BBB Permeability of the first case. Another measurement is the blood-brain permeability-surface area product or CNS Permeability where compounds with a logPS >−2 will be able to enter the Central Nervous System (CNS), while those with logPS <−3 will be unable to penetrate the CNS. For the current study, both cyclopeptides are predicted to do not penetrate the CNS (Pires et al., 2015).

#### 3.2.3 Metabolism

The computed metabolism properties of the Pashinintides A and B are presented in Table 6.

**TABLE 6:** Metabolism properties of the Pashinintides A and B.

Property	Pashinintide A	Pashinintide B
CYP2D6 substrate	No	No
CYP3A4 substrate	No	No
CYP1A2 inhibitor	No	No
CYP2C19 inhibitor	No	No
CYP2C9 inhibitor	No	No
CYP2D6 inhibitor	No	No
CYP3A4 inhibitor	No	No

Cytochrome P450 is an important detoxification enzyme in the body. Many drugs are deactivated by the cytochrome P450 isoforms while some can be activated by it. As can be seen from Table 6, both cyclopeptides are predicted as not being P450 inhibitors for any isoform. It is al important to know if a given compound is likely to be a cytochrome P450 substrate. The predictions indicate that this will be not the case for any of the cyclopeptides (Pires et al., 2015).

#### 3.2.4 Excretion

The computed excretion properties of the Pashinintides A and B are presented in Table 7.

**TABLE 7:** Excretion properties of the Pashinintides A and B.

Property	Pashinintide A	Pashinintide B
Total clearance	0.495	0.856
Renal OCT2 substrate	No	No

Drug clearance occurs as a combination of hepatic clearance and renal clearance (excretion via the kidneys) which is related to bioavailability. The predicted Total Clearance of the Pashinintides A and B are given in log(ml/min/kg) being the value for the former about 55% of the later. OCT2 is a renal uptake transporter that plays an important role in disposition and renal clearance of drugs. In this case, it is predicted that neither of the cyclopeptides will behave as OCT2 substrates (Pires et al., 2015).

#### 3.2.5 Toxicity

The computed excretion properties of the Pashinintides A and B are presented in Table 8.

**TABLE 8:** Toxicity properties of the Pashinintides A and B.

Property	Pashinintide A	Pashinintide B
AMES toxicity	No	No
MRTD	0.136	0.590
hERG I inhibitor	No	No
hERG II inhibitor	No	Yes
ORAT	2.914	2.901
ORCT	3.043	4.044
Hepatotoxicity	Yes	Yes
Skin sensitisation	No	No
*T. Pyriformis* toxicity	0.285	0.285

AMES Toxicity s a widely employed methodology considered to check the mutagenic potential of a given drug using bacteria, thus indicating that when the results is positive, the studied compound will be mutagenic and could behave as a carcinogen. From Table 8, the predictions are negative for both cyclopeptides under study. The maximum recommended tolerated dose (MRTD) provides an estimate of the toxic dose threshold of chemicals in humans. A low value for Pashinintide A and high value for Pashinintide B are found from the results in Table 8. Also, the predictions indicate that both cyclopeptides are unlikely to be hERG I inhibitors, but for the case of hERG II, the behavior will be different: Pashinintide A will not be a hERGII inhibitor while Pashinintide B will. The lethal dosage values (LD50) are a standard measurement of acute toxicity and is defined as the amount of a compound that causes the death of 50% of a group of test animals and are measured through the ORAT and ORCT indices where the predicted values are given in mol/kg.Drug-induced liver injury is a major safety concern for drug development. Hepatoxicity is associated with disrupted normal function of the liver and the predicted values for both cyclopeptides are positive. On the other hand, the predicted values for Skin Sensitisation are negative. T. Pyriformis is a protozoa bacteria whose toxicity is often used as a toxicity test. The predicted values for this parameter are the same for both cyclopeptides (Pires et al., 2015).

### 3.3 Conceptual Density Functional Theory Studies

**FIGURE 4:**
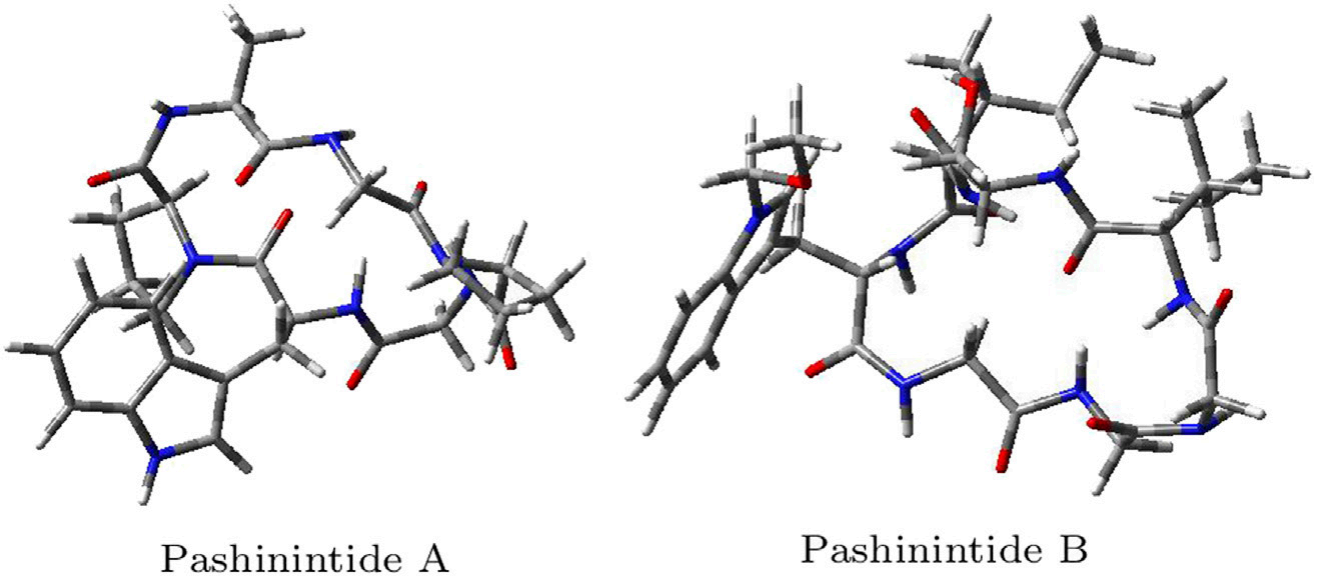
Optimized molecular structures of the Pashinintides A and B.

The optimized molecular structures of the Pashinintides A and B are displayed in Figure 4.

Although the Koopmans-complaint behavior of the MN12SX density functional has been proved previously for the case of marine peptides (Frau et al., 2018; Flores-Holguín et al., 2019a; Frau et al., 2019; Flores-Holguín et al., 2019c; Flores-Holguín et al., 2020; Flores-Holguín et al., 2020a; Flores-Holguín et al., 2020b; Flores-Holguín et al., 2021), we are now performing a further validation for the plant cyclopeptides considered in the present study. This determination has been done by resorting to the in-house developed CDFT software tool and the resulting values are shown in Table 9.

**TABLE 9:** HOMO, LUMO and SOMO orbital energies, HOMO-LUMO gap and the KID descriptors (all in eV) tested in the verification of the Koopmans-like behavior of the MN12SX/Def2TZVP/H2O model chemistry for the Pashinintides A and B.

Molecule	HOMO	LUMO	SOMO	H-L gap	J_*I*_	J_*A*_	J_*HL*_	ΔSL
Pashinintide A	−5.5424	−0.9796	−1.1113	4.5628	0.018	0.054	0.057	0.132
Pashinintide B	−5.6589	−1.2210	−1.3641	4.4379	0.028	0.070	0.076	0.143

As can be seen from the values presented in Table 9, the KID descriptors are all very close to zero meaning that the chosen MN12SX density functional displays a Koopmans-complaint behavior. This in agreement with our previous studies on peptides (Frau et al., 2018; Flores-Holguín et al., 2019a; Frau et al., 2019; Flores-Holguín et al., 2019c; Flores-Holguín et al., 2020; Flores-Holguín et al., 2020a; Flores-Holguín et al., 2020b; Flores-Holguín et al., 2021), thus justifying the adequacy of the MN12SX/Def2TZVP/H2O model chemistry for the purpose of this research.

The results for the global reactivity indices were estimated by making use of the mentioned CDFT tool and are presented in Table 10.

**TABLE 10:** Global reactivity descriptors (in eV) for the Pashinintides A and B.

Molecule	*χ*	*η*	*ω*	S	N	*ω* ^−^	*ω* ^+^	Δ*ω* ^±^
Pashinintide A	3.2610	4.5628	1.1653	0.2192	3.2501	4.2463	0.9853	5.2316
Pashinintide B	3.4399	4.4379	1.3332	0.2253	3.1336	4.6637	1.2238	5.8875

As the global hardness *η* can be regarded as a direct measure of the deformation of the electron density and of the chemical reactivity being related to the HOMO-LUMO gap, it can be seen that Pashininitide A will be slightly more reactive than the other cyclopeptide. The electrodonating ability *ω* ^−^ is more important that its electroaccepting power *ω* ^+^ for both cyclopeptides because of their molecular structures. However, after a comparison of the values of *ω* ^−^ and *ω* ^+^ for each molecule, it can be concluded that there are not important differences between them. The electrophilicity *ω* index encompasses the balance between the tendency to acquire an extra amount of electron density by an electrophile and the resistance of a molecule to exchange electron density with the environment Domingo et al. (2016). By studying the electrophilicities of a series of reagents involved in Diels-Alder reactions (Domingo et al., 2002a; Domingo and Sáez, 2009; Pérez et al., 2003), an electrophilicity *ω* scale for the classification of organic molecules as strong, moderate or marginal electrophiles was proposed being *ω* > 1.5 eV for the first case, 0.8 < *ω* < 1.5 eV for the second case and *ω* < 0.8 eV for the last case (Domingo et al., 2002a; Domingo and Sáez, 2009; Pérez et al., 2003). By inspection of Table 10, it can be said that both peptides considered in this study may be regarded as moderate electrophiles. Notwithstanding, the overall chemical reactivity is about the same for both cyclopeptides. This information could be of interest for future studies on the potential therapeutic ability of these compounds.

Besides global reactivity descriptors, their local counterparts have been developed to get an idea of the differences in chemical reactivity between the atoms within the molecule. Among these local reactivity descriptors are the Fukui functions (Parr and Yang, 1989; Chermette, 1999; Geerlings et al., 2003) and the Dual Descriptor (Toro-Labbé, 2007; Morell et al., 2005, Morell et al., 2006; Martínez-Araya, 2012a; Martínez-Araya, 2012b; Martínez-Araya, 2015), which have been defined as: Nucleophilic Fukui Function (NFF) = *f* ^+^(**r**) = *ρ*_*N*+1_(**r**) − *ρ*_*N*_(**r**), Electrophilic Fukui Function (EFF) = *f* ^−^(**r**) = *ρ*_*N*_(**r**) − *ρ*_*N*−1_(**r**), and Dual Descriptor (DD) = Δ*f*(**r**) = ${\left(\partial \;f\left(r\right)/\partial \;N\right)}_{\upsilon \left(r\right)}$, relating the electronic densities of the neutral, positive and negative species.

**FIGURE 5:**
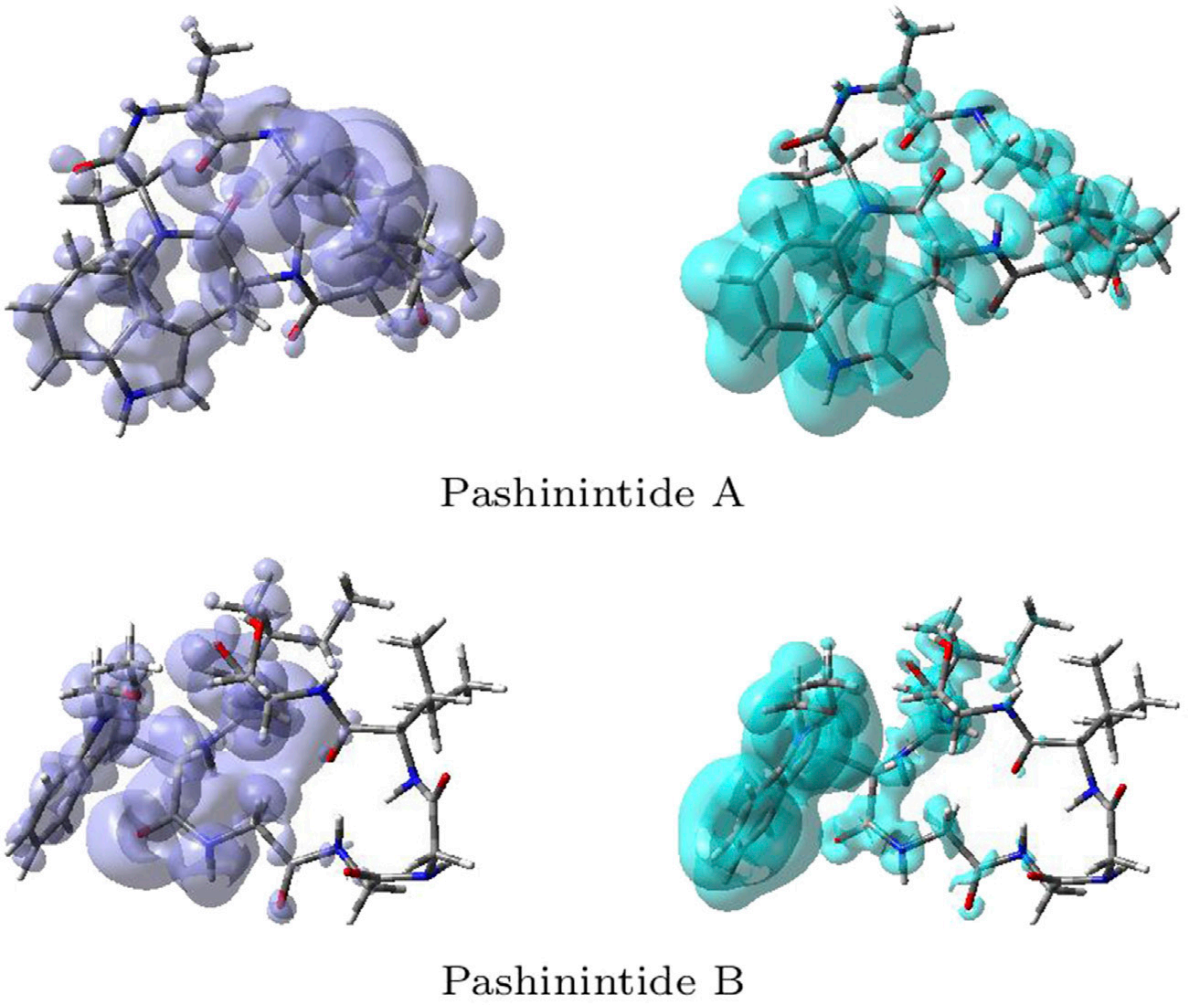
Graphical representations of the dual descriptor DD of the Pashinintides A and B. Left: DD > 0, right: DD < 0.

The NFF, *f* ^+^(**r**), is associated with the sites within a molecular system which are prone to nucleophilic attacks while the EFF, *f* ^+^(**r**), describes those sites that are more susceptible to electrophilic attacks. Although the NFF and the EFF have been used successfully for the identification of reactive sites, the Dual Descriptor Δ*f*(**r**) or DD, can describe unambiguously nucleophilic and electrophilic sites within a molecule (Martínez-Araya (2015)). A graphical representation of the DD for the Pashinintides A and B cyclopeptides is displayed in Figure 5 showing the zones where DD > 0 and DD < 0.

Although these graphical representations allowed to distinguish the regions within the molecules where the Dual Descriptor will be greater or smaller than zero, it can appreciated that there is some overlap between them. Thus, for a better estimation of these reactivity areas it is worth to determine the values of the Condensed Dual Descriptor (Δf_*k*_) (Morell et al., 2008; Frau and Glossman-Mitnik, 2018d) over the all the atoms (excluding H) in comparison with the condensed versions of the Electrophilicity, that is, the Condensed Electrophilicity (*ω*_*k*_) (Domingo et al., 2002b), and of the Nucleophilicity, being the Condensed Nucleophilicity *N*_*k*_ (Pérez et al., 2009). The resulting values are displayed in Tables 11 and 12 for the Pashinintides A and B, respectively.

**TABLE 11:** Comparison of several reactivity descriptors: condensed electrophilicity *ω*_*k*_, condensed nucleophilicity *N*_*k*_ and condensed dual descriptor Δf_*k*_, over the atoms of Pashinintide A. H atoms are not shown.

Atom	*ω*_*k*_	*N*_*k*_	Δf_*k*_
O (1)	0.0247	0.0307	0.0121
O (2)	0.0132	0.0127	0.0075
O (3)	0.0210	0.0028	0.0168
O (4)	**0.1613**	0.0031	**0.1339**
O (5)	0.0129	0.0204	0.0051
O (6)	0.0257	0.0050	0.0201
N (7)	0.0097	0.0045	0.0093
N (8)	0.0576	0.0002	0.0481
N (9)	0.0052	0.0039	0.0033
N (10)	0.0016	0.0129	−0.0023
N (11)	0.0103	0.0020	0.0080
N (12)	0.0146	0.0034	0.0112
N (13)	0.0018	**0.2820**	**−0.0773**
C (14)	0.0035	0.0058	0.0013
C (15)	0.0029	0.0056	0.0009
C (16)	0.0028	0.0099	−0.0005
C (17)	0.0035	0.0071	0.0009
C (18)	0.0191	0.0007	0.0157
C (19)	0.0101	0.0007	0.0082
C (20)	0.0123	0.0004	0.0102
C (21)	0.0233	0.0007	0.0193
C (22)	0.0312	0.0083	0.0237
C (23)	0.0059	0.0337	−0.0045
C (24)	0.0091	0.0046	0.0063
C (25)	0.0076	0.0624	−0.0111
C (26)	0.0094	0.0013	0.0075
C (27)	**0.1956**	0.0006	**0.1632**
C (28)	0.0046	0.0012	0.0036
C (29)	0.0003	**0.3993**	**−0.1114**
C (30)	0.0416	0.0022	0.0341
C (31)	0.0061	0.0052	0.0036
C (32)	0.0081	0.0128	0.0032
C (33)	0.0009	0.0893	−0.0242
C (34)	0.0180	0.0019	0.0145
C (35)	0.0033	**0.3996**	**−0.1089**
C (36)	0.0042	0.0016	0.0031
C (37)	0.0016	**0.1249**	**−0.0336**
C (38)	0.0027	**0.2805**	**−0.0761**
C (39)	0.0028	**0.2514**	**−0.0679**
C (40)	0.0026	**0.1608**	**−0.0428**
C (41)	0.0019	**0.2900**	**−0.0794**

**TABLE 12:** Comparison of several reactivity descriptors: condensed electrophilicity *ω*_*k*_, condensed nucleophilicity *N*_*k*_ and condensed dual descriptor Δf_*k*_, over the atoms of Pashinintide B. H atoms are not shown.

Atom	*ω*_*k*_	*N*_*k*_	Δf_*k*_
O (1)	**0.1768**	0.0238	**0.1213**
O (2)	0.0181	0.0091	0.0105
O (3)	0.0034	0.0003	0.0024
O (4)	0.0030	0.0032	0.0012
O (5)	**0.1346**	0.0291	**0.0891**
O (6)	0.0012	0.0430	−0.0115
O (7)	0.0013	0.0006	0.0008
O (8)	0.0150	0.0037	0.0098
O (9)	0.0016	0.0011	0.0015
N (10)	0.0072	0.0009	0.0049
N (11)	0.0539	0.0162	0.0344
N (12)	0.0020	0.0009	0.0012
N (13)	0.0057	**0.2153**	**−0.0580**
N (14)	0.0003	0.0003	0.0001
N (15)	0.0589	0.0121	0.0392
N (16)	0.0009	0.0008	0.0005
N (17)	0.0059	0.0018	0.0038
C (18)	0.0050	0.0014	0.0032
C (19)	0.0196	0.0014	0.0138
C (20)	0.0038	0.0011	0.0024
C (21)	0.0006	0.0003	0.0003
C (22)	0.0344	0.0361	0.0145
C (23)	0.0009	0.0003	0.0005
C (24)	**0.1587**	0.0090	**0.1123**
C (25)	0.0176	0.0647	−0.0059
C (26)	0.0068	0.0012	0.0046
C (27)	0.0072	**0.3991**	**−0.1100**
C (28)	0.0041	0.0016	0.0025
C (29)	0.0005	0.0001	0.0003
C (30)	0.0130	0.0098	0.0066
C (31)	0.0082	0.1020	−0.0235
C (32)	0.0043	0.0008	0.0029
C (33)	0.0037	0.0010	0.0024
C (34)	0.0005	0.0002	0.0003
C (35)	0.0053	**0.1143**	**−0.0292**
C (36)	0.0180	**0.3832**	**−0.0976**
C (37)	**0.1253**	0.0105	**0.0878**
C (38)	0.0026	0.0026	0.0011
C (39)	0.0004	0.0002	0.0002
C (40)	0.0128	**0.2814**	**−0.0720**
C (41)	0.0024	0.0502	−0.0128
C (42)	0.0096	**0.2328**	**−0.0603**
C (43)	0.0006	0.0001	0.0004
C (44)	0.0074	**0.1523**	**−0.0386**
C (45)	0.0114	**0.2742**	**−0.0709**
C (46)	0.0173	0.0022	0.0119
C (47)	0.0006	0.0003	0.0003
C (48)	0.0106	0.0017	0.0072
C (49)	0.0026	0.0009	0.0016
C (50)	0.0009	0.0003	0.0005
C (51)	0.0010	0.0223	−0.0057

Even if every atom within the peptides cannot be graphically individualized due to the large size of the molecules, it is clear from the results in Tables 11 and 12 about which are the sites prone to electrophilic and nucleophilic attacks based on the agreement between the values for the Condensed Electrophilicity *ω*_*k*_ with the positive values of the Condensed Dual Descriptor Δf_*k*_, for one side, and with the values of the Condensed Nucleophilicity *N*_*k*_ and the negative results for the Condensed Dual Descriptor Δf_*k*_.

From Table 11 it can be seen that for Pashinintide A the maximum values for the Condensed Electrophilicity *ω*_*k*_ (shown in bold) are located over the C (27) and O (4) which correlate well with the maximum positive results for the Condensed Dual Descriptor Δf_*k*_ over those atoms. The same situation is found for the case of the Condensed Nucleophilicity *N*_*k*_, whose maximum values over the C (29) and C(35) correlate with the maximum negative values Condensed Dual Descriptor Δf_*k*_ localized on those atoms. The closeness between C(27) and C (29) explains the overlap between the two regions within the graphical representation of the Dual Descriptor.

For the case of Pashinintide B, it can be appreciated from Table 12 that the maximum values are located over the O (1), C (24), O (5) and C (37) (in that order) correlating in agreement with the greatest positive results for the Condensed Dual Descriptor Δf_*k*_, while for the Condensed Nucleophilicity *N*_*k*_ the order of reactivity will be C (27) > C (36) > C (40) > C (45) > N (13) being the same as for those derived from the Condensed Dual Descriptor Δf_*k*_. As for the case of the other peptide, the partial overlapping between the different reactive areas could be attributed to nearness between C (24) and C (27).

## 4 Conclusion

Two cyclic peptides, Pashinintides A and B, isolated from a terrestrial plant have been studied by resorting to some techniques of common use in the process of drug discovery and development through our proposed Computational Peptidology methodology showing that these kind of molecules can be regarded as potential therapeutic drugs.

With the further objective of analyzing their bioactivity, some useful parameters for future Structure Activity Relationships (SAR) research for the development of therapeutic drugs, their predicted biological targets, and the ADMET (Absorption, Distribution, Metabolism, Excretion and Toxicity) parameters related to the bioavailability and pharmacokinetics of the two plant cyclopeptides under study were predicted and analyzed.

The chemical reactivities of the studied cyclopeptides have been exhaustively analyzed through the optimization of their structures using the DFTBA methodology and the estimation of their electronic properties making use of the MN12SX/Def2TZVP/H2O model chemistry already considered in previous research for the study of peptides, thus verifying its usefulness for this kind of calculations and supplemented with the calculation the Conceptual DFT-derived global and local reactivity descriptors, allowing to identify the preferred reactivity atoms within the molecules.

## Data Availability

The raw data supporting the conclusions of this article will be made available by the authors, without undue reservation.
